# An *in situ* study on the coalescence of monolayer-protected Au-Ag nanoparticle deposits upon heating

**DOI:** 10.1186/1556-276X-9-438

**Published:** 2014-08-27

**Authors:** Jenn-Ming Song, Wei-Ting Chen, Kun-Hung Hsieh, Tzu-Hsuan Kao, In-Gann Chen, Shang-Jui Chiu, Hsin-Yi Lee

**Affiliations:** 1 Department of Materials Science and Engineering, National Chung Hsing University, Taichung 402, Taiwan; 2 Department of Materials Science and Engineering, National Dong Hwa University, Hualien 974, Taiwan; 3 Department of Materials Science and Engineering, National Cheng Kung University, Tainan 701, Taiwan; 4 National Synchrotron Radiation Research Center, Hsinchu 300, Taiwan

**Keywords:** Self assembled monolayer, Alloyed nanoparticle, Coalescence, *In situ* synchrotron radiation X-ray diffraction

## Abstract

The structural evolution of thiolate-protected nanoparticles of gold, silver, and their alloys with various Au/Ag ratios (3:1, 1:1, and 1:3) upon heating was investigated by means of *in situ* synchrotron radiation X-ray diffraction. The relationships between the coalescence and composition of nanoparticles, as well as the surfactant reactions, were clarified. Experimental results show that there existed a critical temperature ranging from 120°C to 164°C, above which the tiny broad X-ray diffraction peaks became sharp and strong due to particle coalescence. The coalescence temperatures for alloy nanoparticle deposits were clearly lower than those for pure metals, which can be ascribed to the rivalry between the thermodynamic effect due to alloying and the interactions between surface-assembled layers and the surface atoms of the nanoparticles. The strong affinity of thiolates to Ag and thus complex interactions give rise to a greater energy barrier for the coalescence of nanoparticles into the bulk and subsequent high coalescence temperature. The influences of particle coalescence on the optical and electrical properties of the nanoparticle deposits were also explored.

## Background

One of the important applications of nanomaterials metallic nanoparticles (NPs) is to manufacture fine-pitch electrical line patterns for organic transistors, radio frequency identification (RFID) antennas, or ultra*-*large*-*scale integration (ULSI) interconnections not only because of the high electrical conductivity and flexibility in handling, but also the low processing temperature [1,2]. The reduced processing temperature is due to the large surface-to-volume ratio of the particles leading to a dramatic lowering of the melting point and sintering transition. Unlike copper NPs which suffer from easy oxidation, the NPs of noble metals, gold and silver, are stable and widely used in the aforementioned interconnect applications [3-9]. However, the price of gold is high, while silver tracks are plagued by electrochemical migration. Strategies such as alloying and core-shell structure have been proposed to achieve better performance. Nanoalloys of gold and silver metals, which have attracted much attention due to high catalytic activities and unique optical properties [10-13], exhibit essentially identical lattice constants and are completely miscible [14], presenting new opportunities for the development of interconnect materials [15-17].

With respect to ligand-protected NPs, the protect shell must be thermally or chemically eliminited, and the NPs need to join together to form continuous conductive networks in order to generate electrical conductance [18]. Coalescence of gold nanoparticles has been studied by means of simulation, surface plasmon resonance absorption, and thermogravimetric analysis [18-21]. Recently, synchrotron X-ray radiations, powerful probing sources to study the structural, physical, and chemical properties of nano-materials [22], were applied to study the morphological and phase transitions of NP deposits [23,24]. Using synchrotron radiation X-ray diffraction (SR-XRD) and small-angle X-ray scattering (SAXS), Ingham et al*.*[24] proposed the mechanisms of coalescence; in sequence, they are desorption or melting of the capping ligands, aggregation of nanocrystals, necking of particles, and subsequent grain growth. However, there is still a lack of insight regarding the alloying effect on the coalescence of NPs.

In this report, a real-time and systematic study into the coalescence of binary gold-silver alloy NPs was performed. The phase evolution upon heating of thiol-protected NPs of gold, silver, and their alloys with various Au/Ag ratios (3:1, 1:1, and 1:3) was monitored by synchrotron radiation XRD. The interactions between ligands and surface atoms of alloy NPs as well as their influence on the coalescence and related properties were investigated.

## Methods

The preparation of the octanethiolate-stabilized gold-silver alloy nanoparticles followed a modified two-phase protocol proposed by Murray [25], which has been described in a previous work [26]. The nanoparticles were synthesized with varying initial Au/Ag molar ratios (0:1, 0.25:0.75, 0.5:0.5, 0.75:0.25, and 1:0) and designated as Au, Au_3_Ag, AuAg, AuAg_3_, and Ag, respectively.

The UV-visible spectra of the nanoparticle solutions were measured by a spectrophotometer (Varian Cary 100 UV-Visible spectrometer, Palo Alto, CA, USA) with a 10-mm quartz cell. A transmission electron microscope (FEI-TEM, Philips Technai G2, Amsterdam, Netherlands) with an accelerating voltage of 200 kV was used to observe the morphology of the NPs and the particle size was measured using Scion Image 4.0.2 image analysis software.

NPs were suspended in tolune solvent with the proportion of 20% by weight. The suspensions were dropped on 5 × 5 mm^2^ Si wafers with a native oxide layer on the surface, which were ultrasonically cleaned in alcohol and acetone, and then dried in an oven at 50°C for 30 min. Each NP deposits/substrate combination was prepared by pipetting NPs suspensions (approx. 30 ± 0.9 μL) onto the substrates with subsequent spin-coating at 500 rpm for 3 s and then 2,000 rpm for 15 s. *In situ* high-temperature synchrotron radiation X-ray diffraction (SR-XRD) was performed at the wiggler beamline BL-17B1 of the National Synchrotron Radiation Research Center (NSRRC), Hsinchu, Taiwan. The incident X-rays were focused vertically by a mirror and monochromatized to 8 keV (*λ* = 1.5498 Å) by a Si(111) double-crystal monochromator. In this experiment, two pairs of slits positioned between sample and detector were used, which provided the typical wave vector resolution in the vertical scattering plane of about 0.003 nm^-1^. The temperature-dependent XRD patterns of all the samples were collected on a resistive heating copper stage at a heating rate of 5°C/min in air. To minimize the collection time, the patterns were collected only in the 33° to 43° 2θ range back and forth at a scan rate of 5°/min and the evolution of the diffraction peaks was monitored simultaneously. The surface morphology observations were performed by scanning electron microscopy (SEM, JEOL JSM-6460, Akishima-shi, Japan). The chemical valence states of the elements on the surface of the NP deposits were examined using X-ray photoelectron spectroscopy (XPS) with Al sources.

To evaluate the electrical performance of the NP deposits, four-point probe measurement of the deposit resistivity after being heated to different temperatures was performed. The corresponding optical absorption properties were also examined using a UV-vis spectrophotometer.

## Results and discussion

### Characteristics of nanoparticles

**Figure 1:**
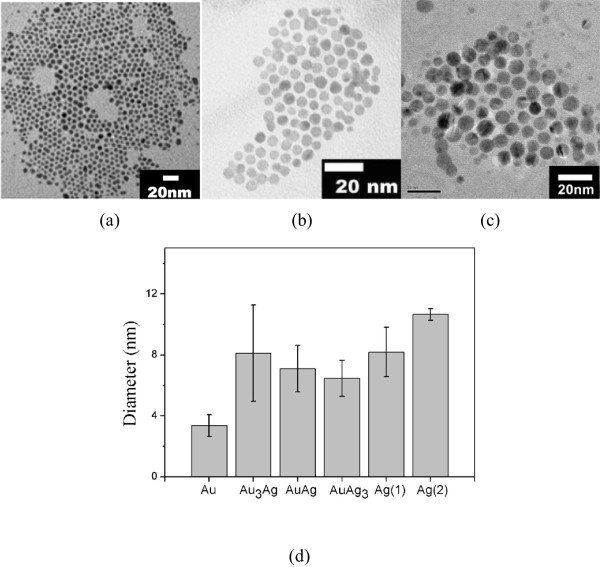
TEM images of nanoparticles (a) Au, (b) AuAg3, and (c) Ag, and (d) core diameters of the nanoparticles used.

**Figure 2:**
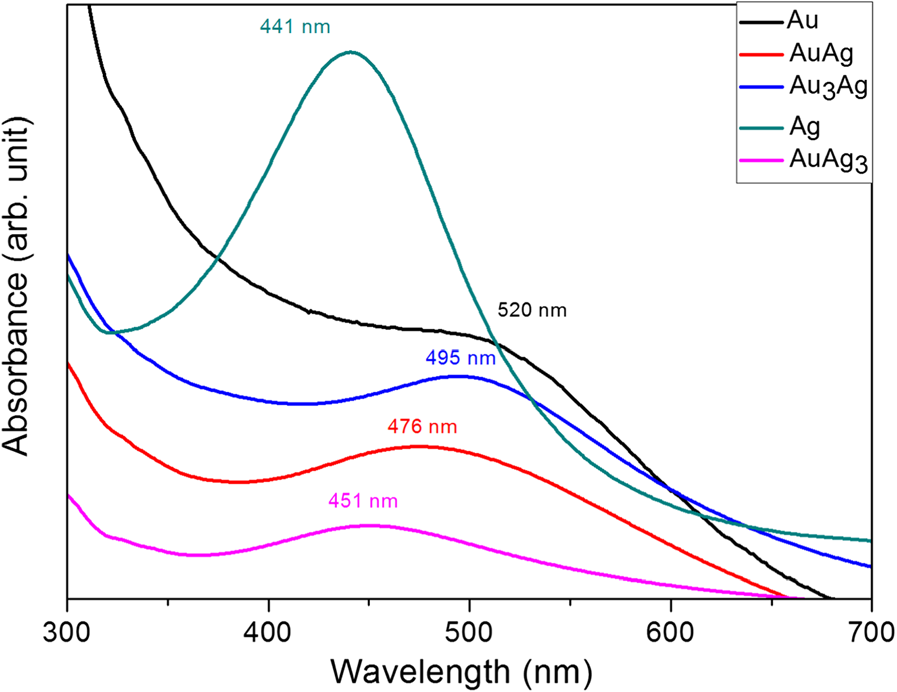
UV-visible absorption spectra of the nanoparticles.

If we take the Ag, AuAg_3_, and Au nanoparticles as examples, the TEM micrographs of the as-prepared thiol-protected nanoparticles (Figure 1a,b,c) show a close-packed arrangement. As revealed in Figure 1c, some of nanoparticles are heavily twinned. Quantitative data given in Figure 1d indicate that the average core diameter of the nanoparticles was 3.6 nm for Au, 8.1 nm for Au_3_Ag, 7.1 nm for AuAg, and 6.5 nm for AuAg_3_. Two batches of Ag NPs were prepared and the particle diameters were 8.2 and 10.7 nm, respectively. The compositional feature of the NPs can be identified from the absorption spectra shown in Figure 2. The alloy formation is inferred from the fact that the optical absorption spectrum shows only one plasmon band. As illustrated, the absorption peak was 520 nm for Au NPs. The plasmon band is blue shifted with an increasing content of silver, and then reached 441 nm for Ag NPs. This tendency is identical to those reported in the literature [27-30].

### Phase transition of nanoparticle deposits upon heating

**Figure 3:**
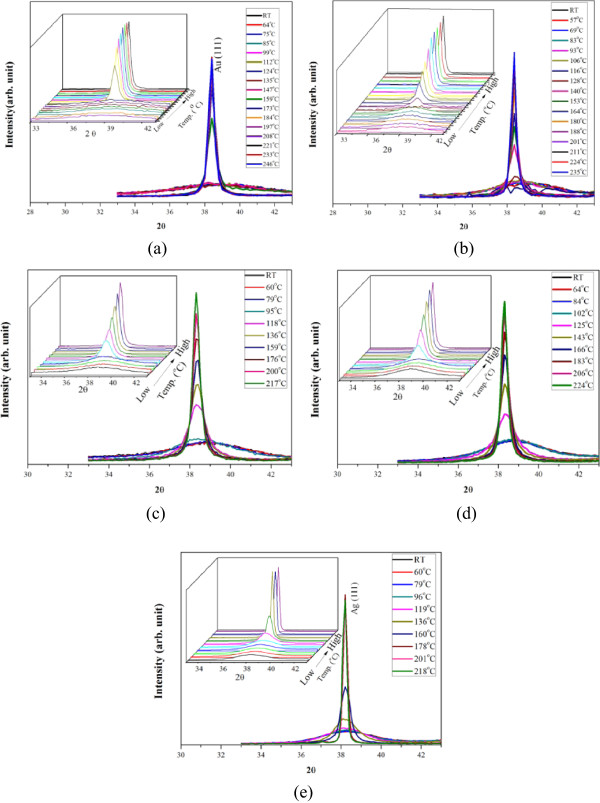
**The evolution of (111) diffraction peak of the NP deposits with respect to heating temperature. (a)** Au, **(b)** Au_3_Ag, **(c)** AuAg **(d)** AuAg_3_, and **(e)** Ag (the X-ray wavelength *λ* = 1.5498 Å).

**Figure 4:**
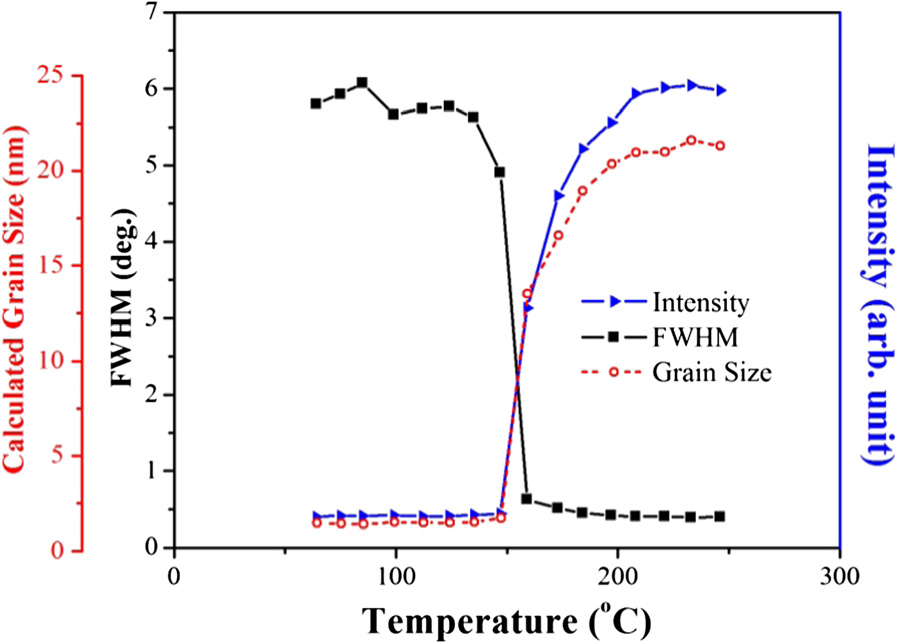
**The intensity, width and calculated grain size of Au(111) peaks with respect to heating temperature.** (Based on the data obtained from Figure 3a).

**Figure 5:**
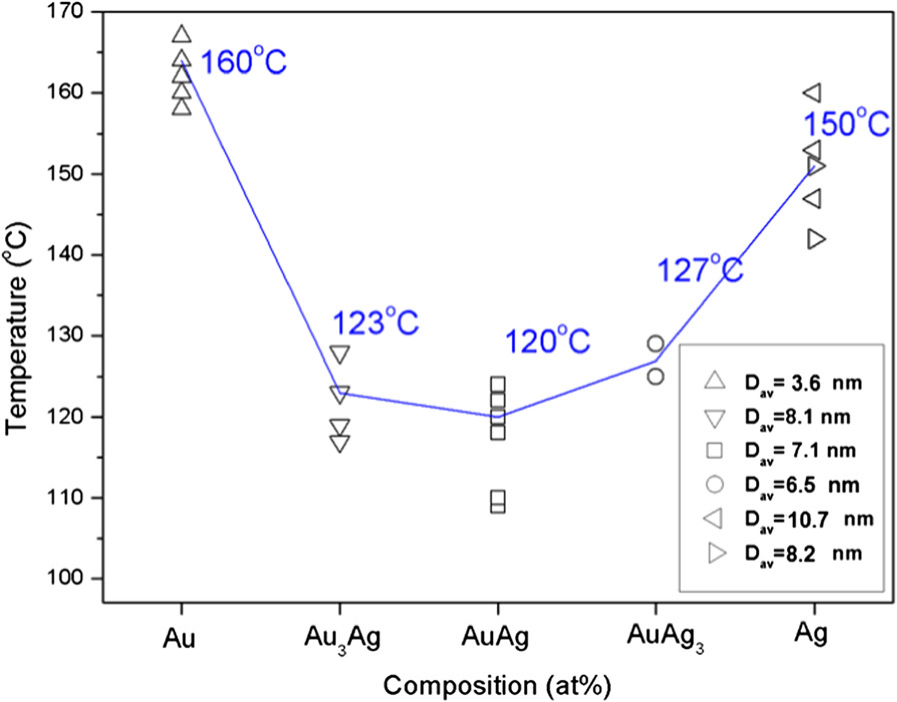
Variation in the coalescence temperature with respect to particle composition.

**Figure 6:**
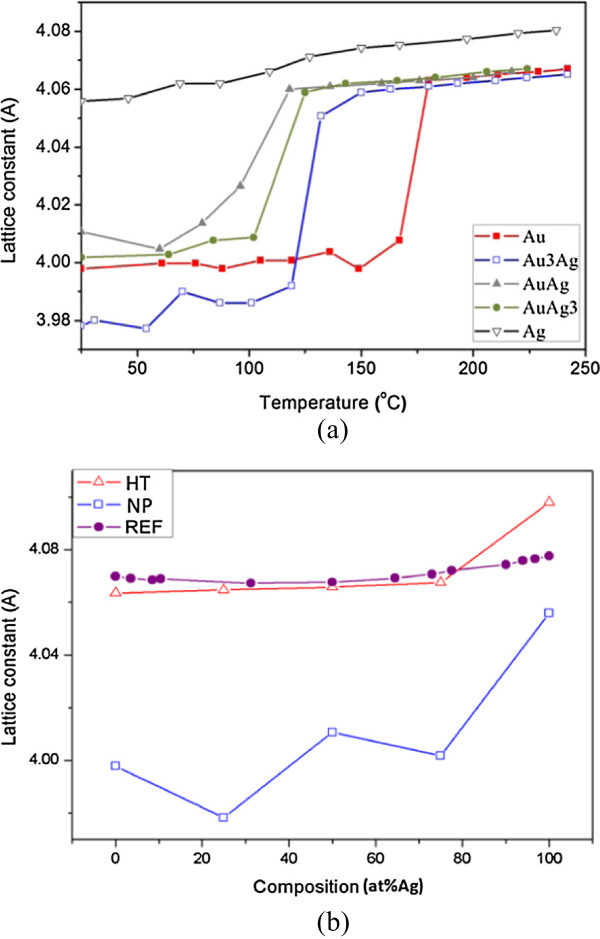
**Lattice constant as a function of heating temperature and size states. (a)** Variation in the estimated lattice constant as a function of heating temperature, and **(b)** Lattice constants at nano and heat-treated states (designated as NP and HT, respectively) compared with the theoretical values (REF).

The SR-XRD patterns of NP deposits measured from 25°C to 250°C are illustrated in Figure 3. It is apparent that broad and weak (111) diffractions appeared at low temperatures due to the size-broadening effect. Taking the Au NPs as example, the quantitative data shown in Figure 4 depict that when the NPs were heated to a critical temperature, the intensity (the maximum peak amplitude) of the broad peak skyrocketed dramatically, and after that, it increased gradually. Figure 4 also illustrates the peak width (full width at half maximum, FWHM) and thus grain size calculated using Scherrer equation given below [31].

(1) $$D=\frac{0.9\lambda }{\beta \cos\theta }$$

where *D* is the grain size, *λ* is the wavelength of the X-ray, *β* is the full width at half maximum, and *θ* is the angle corresponding to the peak. It can be found that the variation in peak width is just opposite to the tendency of increasing intensity. The critical temperature for particle coalescence can be defined as the temperature for the sudden increase in peak intensity, which represents the linking of nanoparticles and a high degree of crystallization [23,24,32,33]. As also indicated in Figure 4, grain growth occurs right after the coalescence of NPs.The coalescence temperature of NP deposits with varying Au/Ag molar ratio are listed in Figure 5. For each sample, the variation in the coalescence temperature was 10 ~ 15°C. The average data show that the coalescence temperature decreased when the Ag content increased from 0 at% (the Au sample, 160°C) to 50 at% (the AuAg sample, 120°C). After that, the coalescence temperature rose and reached 150°C for the samples of 100 at% Ag (the Ag sample, 150°C). This implies that the coalescence temperatures for alloy nanoparticle deposits were significantly lower than those for pure metals. In addition, with respect to the Ag deposits with a small difference in particle size, the coalescence temperatures did not differ too much. The average values are 153.3°C for bigger Ag NPs (10.7-nm diameter in average) and 146.5°C for those with a smaller size (8.2-nm diameter in average).It was also found that the diffractions tended to shift towards low angles due to thermal expansion. The difference in the lattic constants among the deposits was large at room temperature but was reduced significantly when heated to 250°C (Figure 6a). The lattice constants calculated from the diffraction angles for the as-prepared NP deposits and those after being heated to 250°C are illustrated in Figure 6b. In comparison with the annealed bulk alloys (theoretical values), it can be derived that the lattice constants for nanocrystals were about 1.5% smaller than the theoretical values regardless of the composition. Subjected to heating to 250°C, the NP deposits can be regarded as bulk metals judged by their lattice constants.

**Figure 7:**
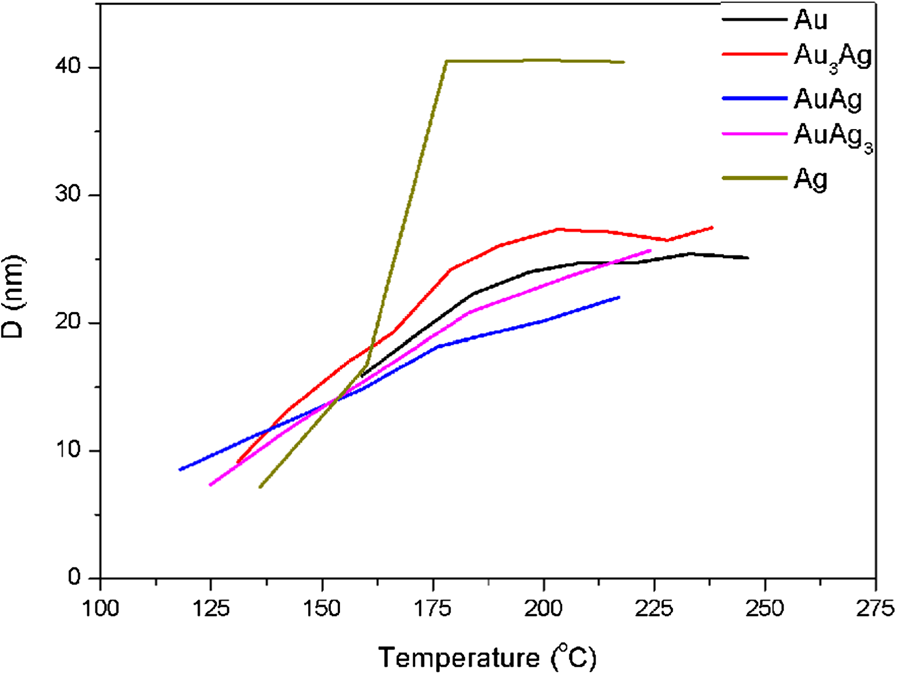
Variations in the estimated particle size.

**Figure 8:**
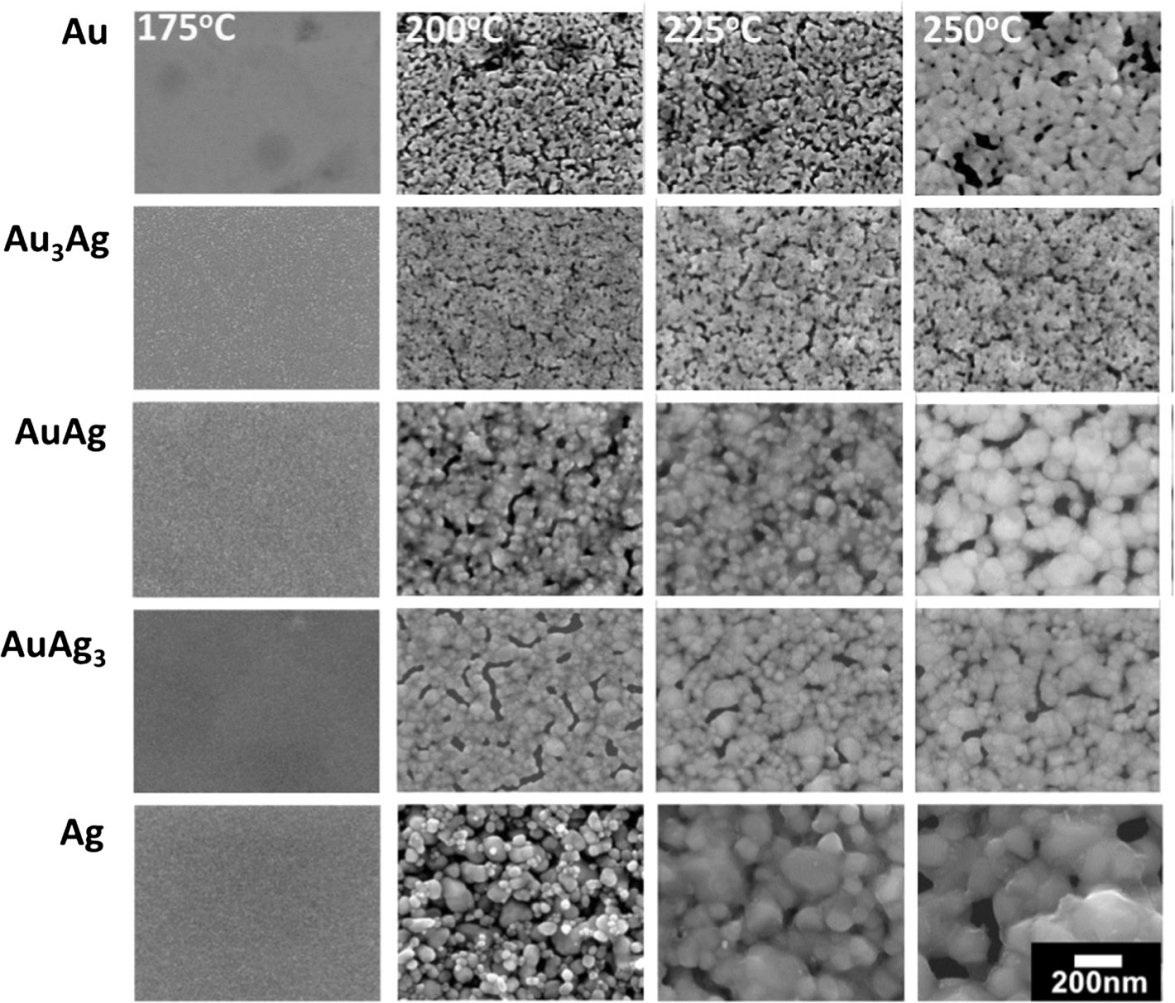
The SEM images of the heat-treated NP deposits.

Using Equation 1, the variations in the estimated particle sizes of all the NP deposits after being coalesced as a function of heating temperature are given in Figure 7. It should be noted that the Scherrer equation assumes the fine particles are strain free. Non-uniform strain causes additional line broadening and gives rise to underestimated crystallite size [32]. This also explains the underestimated diameter for Au NPs prior to coalescence as indicated in Figure 4. Figure 7 illustrates that all the NPs exhibited a particle diameter of about 10 nm at their coalescence temperature. With a higher temperature, the particle sizes of most the NPs increased and reached 20 ~ 30 nm at high temperatures. Remarkably, the Ag NP deposits possessed a greater grain growth rate and the estimated particle diameter after heating to 250°C reached 40 nm, obviously larger than the others. The surface morphologies of the NP deposits after being heated to a specific temperature (Figure 8) verify the extraordinarily large grains of the heat-treated Ag deposits. This should be prevented because discontinuity and even rupture of NP deposits due to abnormal growth have been witnessed [21].

**Figure 9:**
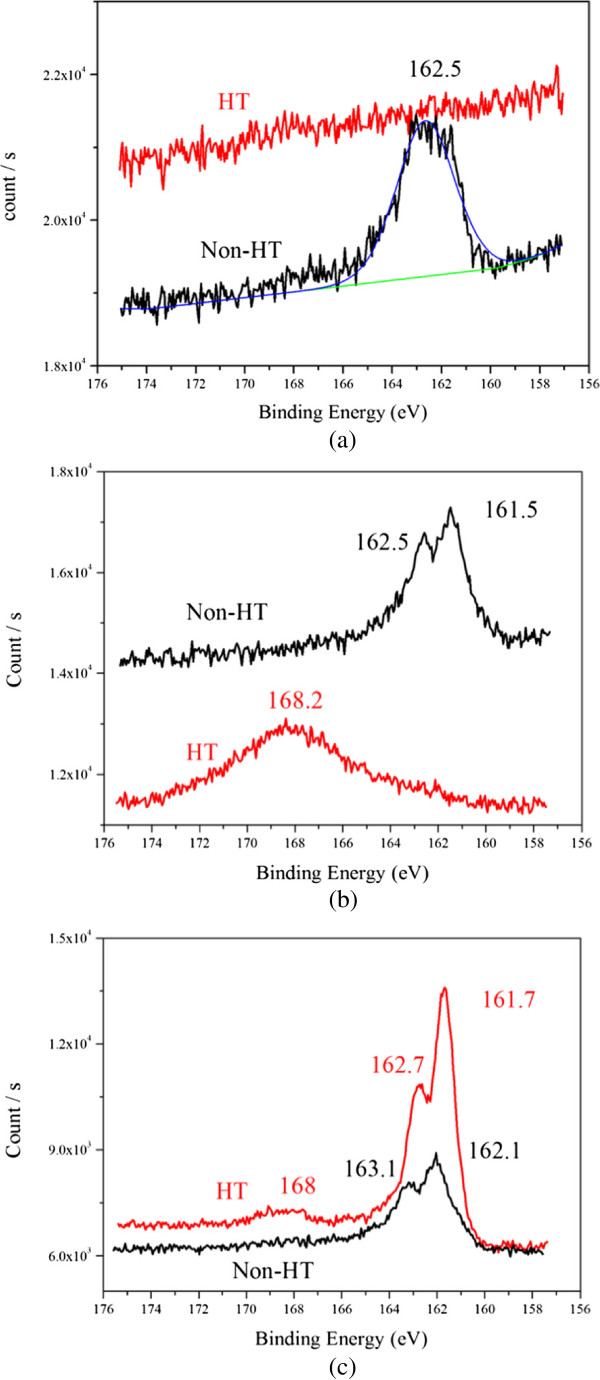
**XPS spectra of the NP deposits (S2p) before and after heating (designated as Non-HT and HT, respectively). (a)** Au, **(b)** AuAg, and **(c)** Ag.

Figure 9a,b,c illustrates the S2p region of the XPS spectra of the Au, AuAg, and Ag deposits under non-heat-treated (Non-HT) and heat-treated (HT) states, respectively. The binding energy values of XPS peaks are also marked. For all the non-heat treated samples, there is a broad peak at 161 ~ 163 eV. This probably consists of two components, the 2p_3/2_ and 2p_1/2_, separated by approximately 1 eV [34]. After heating to 250°C, this broad peak disappeared for the Au and AuAg deposits, but its intensity remained strong for the Ag samples. The other broad peak located at 168 eV was found in the heat-treated Ag and AuAg deposits. It corresponds to S bonded three O atoms and has been observed in previous reports indicating thiols experienced photo-oxidation [34,35]. Accordingly, it can be inferred that the interactions between S and Ag were more complicated and stronger than those between S and Au, which resulted in late desorption of thiols from the surface atoms of Ag. S still bonded with the surface atoms of the pure Ag deposits after heating.

### Optical and electrical properties of nanoparticle deposits subjected to heating

**Figure 10:**
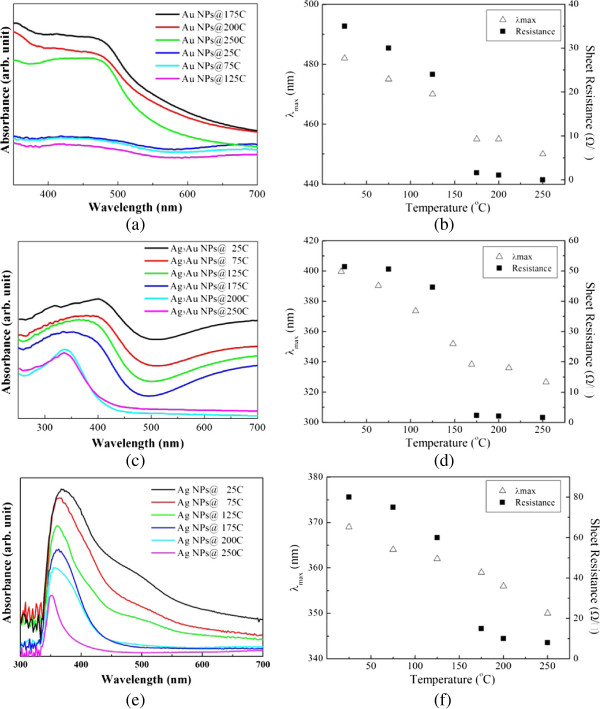
**The evolution of the UV-vis absorbance spectra and electrical resistance.** Absorption spectra of NP deposits after heating at different temperatures for 20 min, and wavelength of absorption peaks as well as corresponding electrical resistance: **(a, b)** Au, **(c, d)** AuAg_3_, and **(e, f) **Ag.

The evolution of the UV-vis absorbance spectra for the NP deposits with respect to the heating temperature and corresponding electrical resistance are illustrated in Figure 10. With a higher temperature, the intensity of the SPR (surface plasmon resonance) absorption curves was suppressed and the absorption bands were gradually blue shifted (Figure 10a,c,e). If we determine the wavelength of absorption bands (*λ*_max_) from the intersection points of the tangent lines of the curves at both sides of the absorption peak, the quantitative data shown in Figure 10b,d,f indicates that there existed a critical temperature ranging from 125°C to 175°C for the change in absorption band and electrical resistance of the NP deposits. Above this temperature range, the absorption peak value and electrical resistance were depressed significantly, resulting from the coalescence of NPs. Two opposite tendencies have been observed regarding the plasmon shift caused by heating of nanoparticles. Anto et al. [18] reported that upon heating to the percolation transition temperature, which was taken to be the mid-point of the insulator-to-metal transition, the plasmon band redshifts and broadens as a mark of the onset of particle coalescence. On the other hand, other research groups found that plasmon bands become narrower and move to the low wavelength end [20,21,36]. Supriya studied the thermal treatment of colloidal Au and suggested that at a lower temperature, the Au colloids aggregate and the high polydispersity of particle size causes broadened plasmon peaks because of the coupling of the interparticle surface plasmons, while at high temperatures, the colloids coalesce and give rise to a narrowing of peak width due to an increase in interparticle spacing or decrease in aggregation [20]. Prevo et al*.*[21] observed the evolution of a uniform, multilayer aggregated nanoparticle structure subject to flame heating. They suggested that a decrease in the average domain size of the metal size results in the spectral blue shift of the SPR absorbance to lower wavelengths. Rast [37] investigated the thermal decomposition of PVP/Ag nanoparticle composite film and observed a decrease in SPR absorbance and blueshifting, which was ascribed to an initial fragmentation of nanoparticle aggregates and subsequent coalescence of NPs due to diffusion.

The SPR results in this study lead us to believe that significant particle coalescence accounts for the remarkable blue shift of plasmon peaks when heating to 125 ~ 175°C. The shrunk surface area contributes to the decrease in absorbance especially for the Au NP deposits. This reveals a faster coalescence kinetics compared with the other two NP deposits containing silver. Figure 10 also demonstrates the sheet resistance shows a consistent tendency with the shift of SPR band, suggesting that the elimination of the interparticle point contact and also the intraparticle grain boundaries reduced electrical resistance [21]. The measured electrical resistivities of the NP deposits for the as-prepared and annealed states are listed in Table 1. It can be found that the resistivity was hugely reduced when subjected to heating due to the removal of the ligand shell on the particle surface and thus particle coalescing. Worthy of notice is that the Ag NP deposits exhibit an inferior electrical resistivity twice as higher as those of Au and AuAg_3_ NPDs. In combintaiton with the above XPS observations, it can be deduced that residual sulfur had a negative influence on electrical conductance.

**Table 1:** Electrical resistivity of the NP deposits

**NPs**	**Electrical resistivity(μΩ-cm)**	
	**As-prepared**	**As-annealed**
Au	1.75 × 10^3^	7.88
AuAg_3_	2.5 × 10^3^	8.32
Ag	3.75 × 10^3^	18.45

### Factors affecting the coalesence of the thiol-protected AuAg nanoparticles

**Figure 11:**
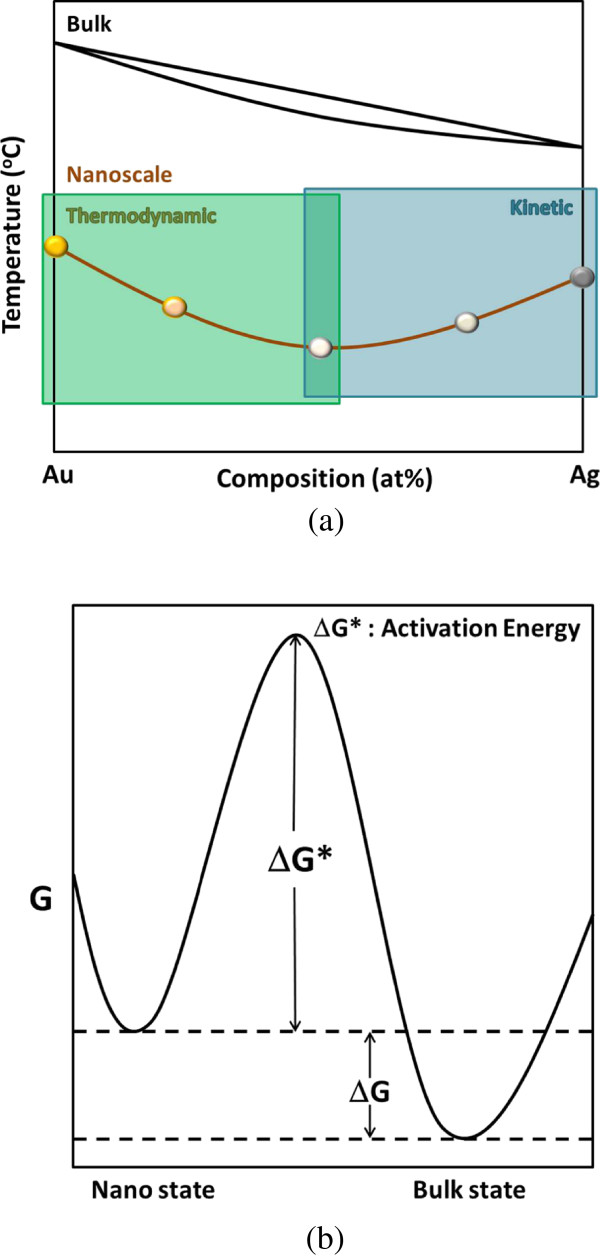
**Transition temperatures of gold-silver alloys and free energy states. (a)** Schematic diagram illustrating the solid-liquid transition temperature of bulk gold-silver alloys and the coalescence temperature of gold-silver alloy NPs against the composition, as well as the dominating factors for particle coalescence, and **(b)** a schematic of Gibbs free energy of metals at different scales, in which ΔG represents the difference in volume free energy and ΔG* is activation energy for particle coalescence.

Particle size has significant influences on the melting and the coalescence of nano-sized particles [19,38-41]. As reported, nanoparticles are characterized by low melting points, low coalescence temperature, and short sintering time as a result of the atom thermal vibration amplitude increase in the surface layer. Although this study focuses on the composition effects, the size-dependent effect on particle coalescence can still be found when two batches of Ag NPs with different diameters are compared. Smaller Ag NPs exhibit relatively reduced coalescence temperature. As for Au NPs with the average diameter of 3.6 nm used in this study, if they have similar size with the other samples (6.5 ~ 10 nm), a higher coalescence temperature is predictable.As mentioned above, the coalescence temperatures of the thiol-capped binary gold-silver alloy nanoparticle deposits followed a convex relation with the silver content as illustrated in Figure 11a, i.e., the average coalescence temperature decreased from 160°C to 120°C at the low silver side, and at the high silver side, it ascended to 150°C for pure Ag NPs. To explain this phenomenon, a rivalry between thermodynamic factors and surface chemistry should be considered.

The joining of nanoparticles begins with the formation of the necks between the particles and is driven by surface atom diffusion [24] or surface melting [19]. If surface diffusion dominates, the higher diffusivity of silver atoms over gold atoms [35] can account for the lower coalescence temperature for the alloy NPDs compared with pure Au NPDs. High diffusivity of silver atoms may also result in a great grain growth rate after particle coalescence and thereby abnormally large grains for the Ag NP deposits. However, the contribution of surface melting should not be neglected. Arcidiacono et al. [19] studied the coalescence of gold nanoparticles and reported that a thin liquid shell due to surface melting may have an important role especially in the early sinter/coalescence stage. Since the transient complete melting of octenthiolate-stabilized Au nanoparticles (with an average diameter of 2.5 + 0.7 nm) at 200°C has been experimentally demonstrated in a recent study [23], a much lower temperature for surface melting can be expected [41-43]. Even though the melting point and latent heat of fusion are dependent upon the particle size, the alloying effect on the solid-liquid transition temperature can still be discussed using the classical thermodynamic equation given below [44].

(2) $$\frac{\partial T}{\partial N_{2}}=-\frac{\left(N_{2}^{\left(L\right)}-N_{2}^{\left(S\right)}\right)T}{\left(1-N_{2}^{\left(L\right)}\right)\Lambda_{1}+N_{2}^{\left(L\right)}\Lambda_{2}}\frac{\partial^{2}G^{\left(S\right)}}{{\left(\partial N_{2}^{\left(S\right)}\right)}^{2}}$$

where *G*^(*s*)^ is the mole free energy of solid phase, Λ_1_ is the latent heat of component 1, Λ_2_ is the latent heat of component 2, *N*_2_ is the mole fraction of component 2, and *T* is the equilibrium temperature of an alloy. Accordingly, the solid-liquid transition temperature in the gold-silver binary system decreases with an increasing silver fraction, and thus, it can be inferred that the coalescence temperature follows the same tendency due to alloying, as marked in the lower left circle (at the low silver side) in Figure 11a.

As to the ascending coalescence temperature at the high silver side, we should consider the ligand shells on the particle surface and their influence on coalescence kinetics, as marked in the lower right square in Figure 11a. A study on ionic monolayer-protected nano-Au and nano-Ag inks by Anto et al. [18] proposed that the coalescence temperature of nanoparticles is not determined by the thermodynamic size melting or by the surface area effect, as previously thought, but by the temperature when a large portion of the dense monolayer is eliminated. In other words, the coalescence temperature depends on the thermal stability and packing density of the shell, rather than the size of the metal core. As reported, the sulfur of octanethiol on Au NPs thermally decomposed at elevated temperatures and the amount was reduced to half of the initial value when heating to around 125°C [45]. This explains why the coalescence of octanethiolate-protected NPs can occur at a low temperature of 120°C.

The above XPS observations demonstrate sulfur remained in silver-rich NP deposits. This is closely related to the chemisorption of thiolates on the surface atoms of gold and silver, which is given as follows [46],

(3) $$R-S-H+M_{n}^{0}\to R-S^{-}M^{+}\cdot M_{n}^{0}+\frac{1}{2}H_{2}$$

where *R* - S^-^ is the absorbing species, thiolates in this case, and *M* represents Au or Ag. Ulman suggested that thiolate monolayers on Ag(111) are more densely packed due to the shorter S…S distance (4.41 Å for Ag(111) and 4.97 Å for Au(111)) [41]. If we take alkanethiolates for example, there are two possible bonding locations for thiolates on Ag(111), i.e., hollow sites and on-tope sites, while thiolates can only be bonded at the hollow sites in the case of Au(111). As illustrated in Figure 11b, it can be deduced that the strong affinity of thiolates for Ag and thus complex interactions gives rise to a greater energy barrier (ΔG*) for the coalescence of nanoparticles into the bulk and subsequent high colescence temperature.

## Conclusions

In this study, the evolution of thiolate-protected binary gold-silver NP deposits with a wide compositional range upon heating in air was studied via *in situ* synchrotron radiation X-ray diffraction and the characteristics of NP deposits before and after heating were investigated. Particle coalescing can be revealed by the sudden intensification of the diffractions, and the coalescence temperature for alloy nanoparticle deposits are clearly lower than those for pure metals. It is suggested that the coalescence of nanoparticles strongly depends on the rivalry between the thermodynamic and kinetic factors, which are respectively due to alloying effect and the ligand/surface atom interactions. Subjected to annealing, gold-silver alloy NP deposits exhibit low electrical resistivity and the ability to avoid abnormal grain growth, showing the great potential as interconnect materials.

## Competing interests

The authors declare that they have no competing interests.

## Authors' contributions

WTC and KHH carried out the main part of synthetic and SR-XRD analytical works. THK carried out the measurement of electrical and optical properties. JMS conceived the research idea, designed the experiments, and prepared the draft. IGC participated in the experimental design and the discussion of the phase transformations. HYL and SJC participated in the SR-XRD analysis. All authors read and approved the final manuscript.

## Authors' information

JMS is a professor with Department of Materials Science and Engineering, National Chung Hsing University, Taichung, Taiwan. IGC is a Professor with Department of Materials Science and Engineering, National Cheng Kung University, Tainan, Taiwan. WTC and KHH are former graduate students supervised by JMS. THK is a former graduate student supervised by IGC and JMS. HYL and SJC are researchers with National Synchrotron Radiation Research Center, Hsinchu, Taiwan.
